# Home-Based Therapy Utilizing Intermittent Manual Compression of the Carotid Artery and Internal Jugular Vein in the Management of Carotid-Cavernous Fistula

**DOI:** 10.7759/cureus.83375

**Published:** 2025-05-02

**Authors:** Subash Phuyal, Biswamohan Mishra, Biswajit Sahoo, Arunprakash Pitchaimuthu, Manoj Kumar Nayak

**Affiliations:** 1 Neuroimaging and Interventional Neuroradiology, Grande International Hospital, Kathmandu, NPL; 2 Neurology, Kalinga Institute of Medical Sciences, Bhubaneswar, IND; 3 Radiology, All India Institute of Medical Sciences, Bhubaneswar, IND

**Keywords:** carotid-cavernous fistula, dilated superior ophthalmic vein, loss of vision, manual intermittent vascular compression, proptosis

## Abstract

A carotid-cavernous fistula (CCF) is an abnormal communication between the carotid artery and the cavernous sinus, which can be of a direct or indirect type. Treatment decisions are based on factors such as the type of CCF, angioarchitecture, severity of clinical symptoms, and risk of vision loss. While most fistulas necessitate endovascular intervention, there are isolated reports of indirect low-flow fistulas resolving with manual vascular compression therapy alone. Herein, we present the case of a 66-year-old female patient who presented with features of indirect CCF exhibiting intermittent headache, orbital swelling, proptosis, and conjunctival chemosis of the right eye, successfully treated with intermittent manual vascular compression therapy, resulting in the complete occlusion of the fistula and the resolution of her ocular symptoms. This case suggests that manual intermittent vascular compression therapy may be a viable non-invasive treatment option for patients with low-flow indirect CCF, potentially obviating the need for early endovascular procedures.

## Introduction

Carotid-cavernous fistula (CCF) is an abnormal vascular malformation that establishes a connection between the carotid arterial system and the cavernous sinus. The most prevalent cause of CCF is direct head trauma, often associated with facial or skull base fractures. Less common etiologies include spontaneous rupture of a cavernous carotid aneurysm, venous thrombosis, and genetic conditions [1]. CCFs can be classified according to various criteria: etiology (spontaneous or traumatic), hemodynamics (high-flow or low-flow), and angioarchitecture (direct or indirect). The direct CCF is defined as direct communication between the internal carotid artery and the cavernous sinus, while the indirect CCF is defined as communication between meningeal arteries and the cavernous sinus [2]. The angiographic classification, which delineates the angioarchitecture of the lesion, is pivotal for devising appropriate therapeutic strategies [3].

The clinical features of CCFs are influenced by their size, location, duration, flow dynamics, and venous drainage pathways and the presence of arterial or venous collaterals. Direct CCFs are usually traumatic in etiology and may demonstrate Dandy's triad, namely, exophthalmos, bruit, and conjunctival chemosis, although not always present [4]. Most patients present with proptosis, chemosis, diplopia, pain, trigeminal nerve dysfunction, elevated intraocular pressure, and vision loss [5]. Intracranial hemorrhage may occur if there is retrograde shunting into the cerebral veins [6]. Indirect CCFs are usually spontaneous and may present with chronic red eyes, proptosis, ocular bruits, and features of glaucoma [2]. We report a case of an indirect CCF that was successfully resolved through manual intermittent vascular compression therapy alone.

## Case presentation

**Figure 1:**
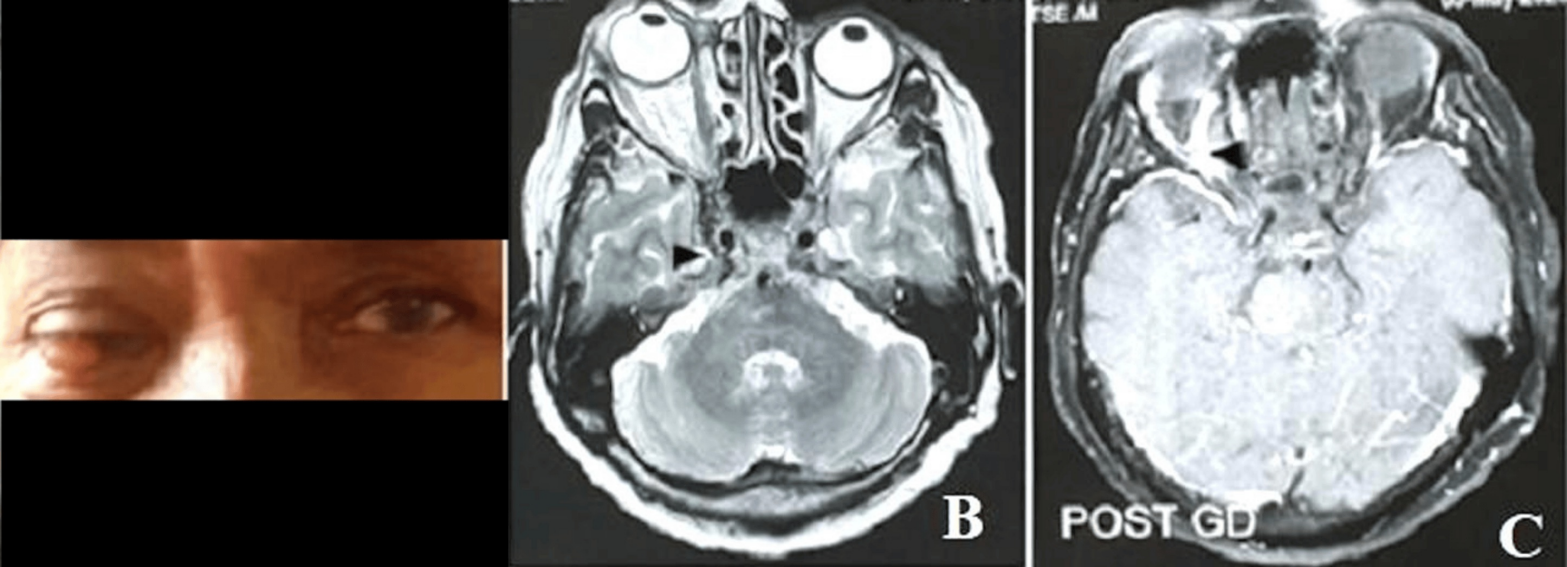
Clinical and MR images of the patient with carotid-cavernous fistula (A) The clinical photograph shows redness and proptosis of the right eye. (B, C) Axial T2-weighted and post-contrast images of the brain showing proptosis of the right eye, bulky right cavernous sinus, and dilated right superior ophthalmic vein. MR: magnetic resonance

**Figure 2:**
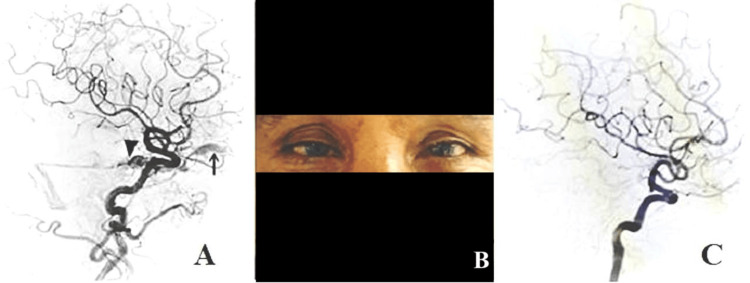
Pre- and post-treatment DSA images and clinical photograph after treatment (A) Right internal carotid artery angiogram (lateral view) shows an early opacification of the cavernous sinus (arrowhead) and early (retrograde) drainage into the dilated right superior ophthalmic vein (black arrow). (B) The clinical photograph shows the resolution of conjunctival redness and proptosis. (C) Right internal carotid artery angiogram (lateral view) at the two-month follow-up shows complete obliteration of the fistula. DSA: digital subtraction angiography

A 66-year-old woman presented with complaints of intermittent headache for one month, orbital swelling, and progressive loss of vision in the right eye for 10 days. She also had diplopia on the lateral gaze. There was no history of head trauma. Her thyroid function was within normal limits (thyroid-stimulating hormone (TSH): 1.5 mIU/L). On examination, proptosis and conjunctival chemosis of the right eye were present (Figure 1A), with a mildly dilated right pupil sluggishly reacting to light. The left eye examination was normal. Contrast-enhanced magnetic resonance imaging (MRI) revealed proptosis of the right eye, dilated right superior ophthalmic vein, and outward bulging of the lateral wall of the right cavernous sinus (Figure 1B, 1C). Multiple tiny collaterals were observed around the right cavernous sinus, extending anteriorly and posteriorly. Diagnostic cerebral angiography revealed an indirect right CCF with arterial feeders from the middle meningeal artery and meningeal branches of the internal carotid artery (Figure 2A).

Venous drainage was directed into the left superior ophthalmic vein, confirming a diagnosis of indirect CCF. Right carotid bifurcation was normal. The patient was informed about the nature of the condition and the available treatment options, including conservative management, neurosurgery, radiosurgery, and endovascular techniques. The patient opted for a trial of manual vascular compression. 

She was instructed to perform manual compression of the ipsilateral common carotid artery for 30 seconds, four to six times a day, either while sitting or while lying down at home. Regular follow-ups included serial examinations, vision tests, intraocular pressure measurements, and fundoscopic examinations. The patient exhibited progressive improvement in symptoms and signs during subsequent follow-ups. After two months, there was a significant reduction in chemosis and complete resolution of proptosis (Figure 2B). Follow-up digital subtraction angiography (DSA) revealed obliteration of the fistula (Figure 2C). She was advised to follow up regularly at two-month intervals for at least 12 months.

## Discussion

Direct CCFs are high-flow fistulas and result from direct communication between the intracavernous carotid artery and the cavernous sinus. Common causes of direct CCFs are ruptured aneurysms and trauma. Indirect fistulas are generally low-flow and indirectly fed by dural feeders from the internal carotid artery, external carotid artery, or both.

The treatment modalities for CCFs include medical management, neurosurgery, radiosurgery, and endovascular repair [7]. Direct fistulas rarely resolve spontaneously [3]. Transarterial or transvenous endovascular repair, using coil embolization or detachable balloons, is generally preferred due to its high success rate [8,9]. In certain cases, solid or liquid embolic agents or stents may be employed in the parent vessel to exclude the fistula from cerebral circulation. However, because direct fistulas involve high flow, there is a risk of distal migration of solid or liquid embolic materials. Recently, endovascular-covered stent grafts have been introduced as an alternative to detachable balloons or coils [10]. In low-flow indirect CCFs, conservative management with intermittent vascular compression can be attempted. The patient will compress the affected common carotid at the neck for 30 seconds many times a day for 4-6 weeks [4]. Some predictors of successful compression therapy include patients with low ocular pressure, a shorter interval between the onset of symptoms and the start of therapy, and venous drainage through the superior ophthalmic vein [11,12]. Compression therapy is relatively contraindicated in patients with cortical venous reflux, known carotid disease, or a history of stroke [9]. Patients who experience deterioration of vision following compression therapy should also discontinue treatment [13].

Compression therapy transiently reduces arteriovenous shunting by decreasing arterial inflow and enhancing venous outflow. This approach promotes spontaneous thrombosis within the cavernous sinus and its branches, ultimately leading to the resolution of the low-flow fistula [13]. Patients should be advised to use their contralateral hand for vascular compression to ensure that if ischemia develops, the symptomatic arm falls away from the neck, thus allowing immediate revascularization of the cortex. Many patients with CCFs can have waxing and waning symptoms, possibly because of cavernous sinus thromboses and re-routing of venous flow in various directions [14]. Sometimes, the CCF can transition to a higher-risk pattern despite apparent clinical improvement. Hence, any change in clinical features should be followed up on promptly and accurately. The "white eye syndrome" is the clinical state where there is an apparent improvement of ocular symptoms because of the spontaneous occlusion of the venous drainage pathways to the orbit [15]. To rule out this possibility, we confirmed the obliteration of the fistula by DSA.

## Conclusions

This report demonstrates the efficacy of intermittent manual vascular compression in managing indirect CCFs, providing an alternative to more invasive endovascular treatments. This approach is likely underutilized and should be considered for patients, particularly in low-resource settings where endovascular therapy may be costly or unavailable. However, it is crucial to ensure the careful and frequent monitoring of clinical progress when using this modality.
